# A hybrid numerical/machine learning model development to improve the bimetal performance in the electric circuit breakers

**DOI:** 10.1038/s41598-022-22763-3

**Published:** 2022-10-27

**Authors:** Abdul Rahman Mallah, Nawaf Aljuraid, Omer A. Alawi, Zaher Mundher Yaseen, Kamaljeet Singh, Adel Ataki

**Affiliations:** 1 Department of Engineering, Reykjavik University, Menntavegur 1, Reykjavík, 102 Iceland; 2 Alfanar Electrical Systems, Riyadh, 11383 Kingdom of Saudi Arabia; 3 Department of Thermofluids, School of Mechanical Engineering, Universiti Teknologi Malaysia (UTM), Skudai, 81310 Johor Bahru, Malaysia; 4 New Era and Development in Civil Engineering Research Group, Scientific Research Center, Al-Ayen University, Thi-Qar, 64001 Iraq; 5 Department of Mechanical and Process Engineering, TU Kaiserslautern,, Kaiserslautern, Germany

**Keywords:** Power distribution, Mechanical engineering, Computational science

## Abstract

Bimetals are widely used as a thermal tripping mechanism inside the miniature circuit breakers (MCBs) products when an overload current passes through the circuit for a certain period. Experimental, numerical, and, recently artificial intelligence methods are widely used in designing electric components. However, developing the bimetal for MCB products somewhat differs from developing other conductor items since they are strongly related to the electrical, mechanical, and thermal performance of the MCB. The conventional experimental and numerical approaches are time-consuming processes that cannot be easily utilized in optimizing the product's performance within the development lead time. In this study, a simple, fast, robust, and accurate novel methodology has been introduced to predict the temperature rise of the bimetal and other related performance characteristics. The numerical model has been built on the time-based finite difference method to frame the theoretical thermal model of the bimetal. Then, the numerical model has been consolidated by the machine learning (ML) model to take advantage of the experiments to provide an accurate, fast and reliable model finally. The novel model agrees well with the experimental tests, where the maximum error does not exceed 8%. The model has been used to redesign the bimetal of a 32 A MCB product and significantly reduce the maximum temperature by 24 °C. The novel model is promising since it considerably reduces the required design time, provides accurate predictions, and helps to optimize the performance of the circuit breaker products.

## Introduction

Miniature circuit breakers are protection devices used to save all the electric circuit elements like devices and wires additional to humans from different kinds of the power faults such as overload currents, short-circuit currents, and other faults^1,2^. MCBs must be equipped with many automated tripping mechanisms to respond to different power faults. The overload tripping mechanism is a mandatory protection system that must be provided in the MCB to protect from the overload currents that exceed the circuit breaker’s nominal current^3,4^. Several methods to protect the circuit from the overload currents, mainly by using thermostatic bimetals^5^ and electronic circuits^6^. Thermostatic bimetals are widely utilized for MCB products since they provide robust and durable operation besides their simple utilization and cheap cost compared to other protection mechanisms. However, selecting the proper composite for the bimetal is a bit complicated process since it requires maintaining a good harmony between the electrical and thermal performances besides providing the proper mechanical functionality by breaking the mechanism at the overload conditions.

The developers follow several methods to obtain the thermal behavior of the electrical elements such as the empirical method^7^, where thermocouples, transient thermal tester and infrared thermometer can be used to precisely obtain the temperature of the current-carrying parts^8^. Additional to the empirical method, mathematical modeling^9^ and numerical approaches are also commonly used to predict the temperature rise of electrical appliances^10–14^. Szulborski et al. successfully utilized a multi-physics numerical method in simulating the temperature rise of the three-phase busbar system used in low voltage switchgear. In contrast, electric–thermal–fluid flow coupled analysis exhibited accurate results when validated with the experimental results^15^. The numerical approaches have also been utilized for simulating the temperature rise of an insulated, high voltage cable, coupling the electric, thermal and fluid flow numerical studies lead to accurate predictions compared to the experiments^16^.

Recently, ML models applications have been observed remarkably in different engineering and sciences diceplines^17–20^. More particularly, ML models have been used widely in developing electric elements^21–25^. The artificial neural network was utilized to select the appropriate busbar material to reduce the temperature rise in the lithium-ion batteries, and the Bayesian Regularization (BR-16) method was found to make accurate predictions of the temperature rise^26^. A data-driven neural network approach with a nonlinear autoregressive network has been utilized to predict the lithium-ion battery's temperature for fast charging and efficient running of the cooling system^27^. A novel method was followed by combining the data-driven analysis and artificial neural network method and applied to obtain the temperature rise of the internal contact inside the gas-insulated switchgear during the overheating fault. The novel approach reduces the accumulated error and improves the prediction^28^. More related studies^29–34^, are listed in Table 1.

**Table 1:** Recent studies of electrical distribution systems.

Authors, Year	Application	Methodology	Comments
Smirnova et al., 2015^25^	Laminated busbar systems used in power electronics	1. Analytical (lumped parameter thermal model), 2. Numerical (FEM) 3. Empirical	Strengths: both analytical and numerical approaches give an accepted accuracy in predicting the temperature rise of the laminated busbars. The error does not exceed 4.7% Weaknesses: busbars systems are straightforward, so, the advantages of analytical and FEM methods cannot be generalized to complex systems like MCBs
Delgado et al., 2017^26^	Low voltage bus duct system	1. Numerical 2. Empirical	Strengths: the numerical model validated with the experimental measurements with an error below ± 4% Weaknesses: busbars systems are elementary, so, the advantages of the numerical method cannot be generalized to complex systems like MCBs
Laib dit Leksir et al., 2018^27^	Electrical equipment	ML (support vector machine method)	**Strengths:** analyzing infrared thermal images and implementing the support vector machine method results in accurate fault predictions. The error was found to be lower than 2% **Weaknesses:** ML approach needs collecting a lot of experimental data, and so, a pretty large time for that, which makes ML not favorable for industrial product design and development projects
Molitor et al., 2020^28^	Laminated busbar in a low voltage switchgear	Numerical	Strengths: the accuracy of the simulation is accepted for industrial product development purposes. The simulation error reaches 7.1% Weaknesses: busbars systems are very simple, so, the advantages of the numerical method cannot be generalized to complex systems like MCBs
Mary & Sugumaran, 2020^29^	High voltage nano-coated busbar	1. Analytical 2. Numerical (magneto-thermal-structural multiphysics)	Weaknesses: there are discrepancies in the results obtained analytically and numerically. The difference between the analytically obtained deformation and the numerical result exceeds 27%. Further, there is no reference study to estimate the error of the study methodology
Gangadhara Roa et al., 2021^30^	Class B and Class F compact busbar system	Numerical	The numerical model was utilized in obtaining the temperature distribution and the heat transfer coefficient when applying different insulation materials Weaknesses: busbars systems are very simple, so the numerical method's advantages cannot be generalized to complex systems like MCBs. There is no reference study to estimate the error of the study methodology

Computational fluid dynamics (CFD) modeling of the water-cooled heat sink used in a solid-state circuit breaker could predicting the thermal performance and temperature rise with a small error^35^. However, there is a lack of technical reports about the temperature rise of thermostatic bimetals inside the circuit breaker products^36^.

For the circuit breaker products that have dozens of components assembled within a compact construction, simulating the temperature rise of the current-carrying parts is a bit sophisticated process due to the interlaced and correlated interference of thermal and mechanical behaviors. The bimetal bends when the current passes through the circuit due to its temperature rise. At a specified overloading current, the bimetal must be capable to bend and apply the required force for tripping the switching mechanism. The overload current at which the overloading protection mechanism trips the breaker is defined by different standards, for instance, IEC 60898-1 standard indicates the MCB shall not break for a current $\le\, 1.13 \, \times\, {I}_{n}$. When the overloading current exceeds $1.13\,\times\, {I}_{n}$ and is still below $1.45\,\times\, {I}_{n}$, the overload protection mechanism must break within a period that does not exceed 3600 s. Furthermore, the temperature rise of the terminals must not exceed 60 °C, and 40 °C for the breaker’s handle when the MCB operates at ${I}_{n}$ conditions. The requirements of the IEC 60898-1 standard are discussed in “Experiment”. The bimetal plays a role in determining the maximum temperature of the specified parts during different operating conditions since it has a relatively lowest electric conductivity compared to other current-carrying parts. Developing the bimetal for MCB products must consider the criteria mentioned above. Furthermore, the development of MCB needs to cover a broad spectrum of products with rated currents ranging starting from 6 to 125 A, There are tens of bimetal composites that are commercially available for MCB applications. Besides that, there is a vast range of the MCB’s rated currents. It is required to test plenty of bimetals for each rated current to choose the one that complies with the requirements of the regulatory standard. Additionally, sometimes it is required to change the bimetal dimension, which leads to performing more experimental rounds. Designing the bimetal mechanism for MCB products requires hundreds of experimental iterations, which is a significantly time-consuming process. Furthermore, the development cost is high since it intensively utilizes the assets and human resources in the industry.

Using numerical methods like FEM or CFD requires tens or even hundreds of iterations considering multiphysics phenomena (electro-thermal, fluid flow, thermal conduction, convection and radiation, etc.). The MCB is composed of tens of parts made of different materials and packed in a small space, which complicates the numerical simulation to get relatively accurate predictions. The complexity of the MCB product makes the simulation task not so easy and so, extra attention is required to build a numerical model capable of predicting the thermal behavior with acceptable accuracy. For that, the numerical methods are also time-consuming, with few reports of their accuracy in the literature. To overcome the limitations in available methods, a hybrid, numerical approach augmented by the ML model is suggested in this study:

- Numerical model based on theoretical heat transfer model: forms the correlations between the different factors affecting the thermal behavior of the bimetal inside the MCB like the materials' thermos-physical properties, bimetal dimensions, etc.
- ML model: to only calculate the correction factors of the theoretical model since the primary relation of physical characteristics is already formulated. Hence, relatively few numbers of the training samples are quite enough to perform the ML calculations.
- After obtaining the correction factors, the NM can be efficiently utilized to predict the bimetal's temperature rise. Because NM is composed of a single element, the solution is significantly fast. In fact, the solution can be performed within a few seconds.

This study presents a novel, fast and robust method for developing a cantilever thermostatic bimetal to protect from the overload current. A simple time-based finite-difference model was established to set the theoretical frame of the bimetal thermal and mechanical behavior. Then, an ML model was employed to exploit the experimental results to consolidate the theoretical model and improve its accuracy. The augmented numerical model by machine learning technique (NMML) has been validated and then used to improve an MCB product's performance. The theoretical frame reduces the number of parameters in the model so that the number of training samples required for the ML is less. Furthermore, the theoretical model provides more details about temperature development during the operation. The novel NMML model exhibits a promising potential for solving engineering problems; the accuracy of the novel model and the fast solution are both important factors for developing engineering solutions where the product reliability and the development lead time have great considerations.

## Methodology

### Thermal tripping mechanism for MCB

Cantilever bimetals are widely used in MCBs because of their simple construction and robust functionality. There are two ways to use the bimetals in the MCB products; the first one is as an active current-carrying part in the circuit breaker, so, the current passes through the bimetal, leading to an increase in its temperature and it correspondingly deflects. This is called direct heating and is used for relatively low-rated current MCB. The second way of operating is by linking the bimetal into a current-carrying part in the MCB, so, it is indirectly heated by the live metal part, but the current does not pass through it. This is called indirect heating, where it is used for higher-rated currents. The bimetals are composed of high expansion and low expansion components. The two components are iron, manganese, and nickel composites, with the optional existence of copper, chromium, and cobalt to adjust the mechanical, electrical, and thermal properties^37^. Nevertheless, all other current-carrying parts in the MCB are mainly made from copper, whereas steel is used for the terminals. That makes the bimetal one of the highest electrical resistance components inside the MCB, and so, it generates a relatively high amount of heat and has a higher temperature inside the circuit breaker (for direct heating bimetal).

The thermostatic bimetal is usually mounted beside the primary switching mechanism in the MCB with maintaining a designated clearance. The temperature of the bimetal increases proportionally with increasing the current that passes through the circuit. The bimetal responds to the temperature rise by deflecting toward the main mechanism. This deflection increases with the temperature rise closing the gap with the main mechanism. At a specified current and its corresponding temperature, the bimetal closes the clearance with the main mechanism and applies the required force to break the main mechanism and trip the MCB.

Designing the direct heating bimetal for the MCB is not a simple process since it is required to obtain its temperature for different electric currents. Consequently, its deflection can be calculated based on the temperature rise. Many factors play a significant role in determining the temperature rise of the bimetal, mainly its size, and thermal and electrical properties besides the surrounding components, which affect the conduction, convection, and radiation heat transfer.

For the MCB products, the behavior of the bimetal is firmly correlated to the rate of electrical current of the MCB, which is mainly based on the application. Therefore, the standards are maintained to have a specific temperature due to heat generated from the current flow through of the bimetal to get the desired instantaneous deflection to obtain the breaking current under temperature rise conditions from the application's point of view.

**Figure 1:**
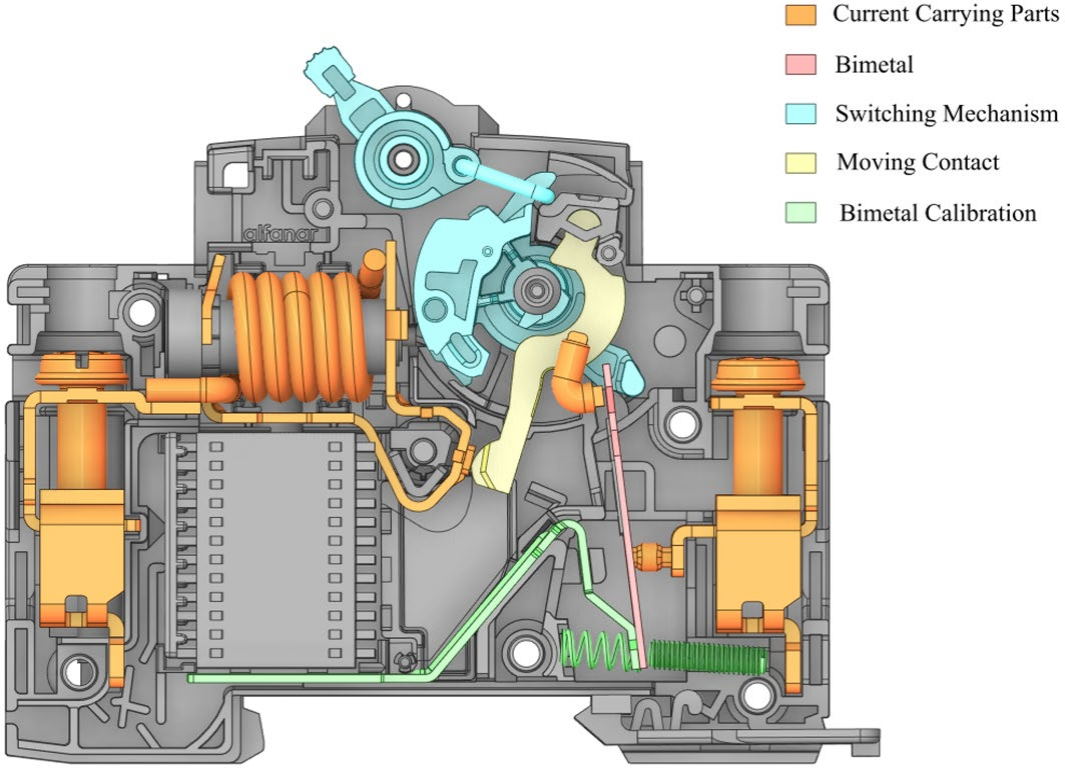
Illustration of the branch circuit breaker highlighting the bimetal, the switching mechanism, and the components used for calibration of the bimetal, genegrated using “ANSYS Spaceclaim R2 2021”^38^ (https://www.ansys.com/products/3d-design/ansys-spaceclaim).

The purpose of the bimetal in the MCBs is to trip the breaker in high thermal conditions (overload conditions). Therefore, the purpose of deflecting at a certain distance with a specific temperature in a period means it is a function of temperature and time. The mechanism work of the bimetal will be started after switching the MCBs to the ON position which will move the moving contact to be connected with the fixed contact (current-carrying parts) to be in a closed-loop position as it is illustrated in Fig. 1 “genegrated using ANSYS Spaceclaim R2 2021”^38^, which will lead to allowing the breaker to carry the current from the inlet to outlet terminals (cable in and cable out). Hence, as the current passes the parts of the breaker, there will be a heat generation due to the resistivity of the parts, and in some cases, if the current is more than it should be, then consequently the temperature will increase inside the breaker which will cause a temperature rise. As a result, the bimetal will deflect due to that rise in the temperature until it touches and pushes further the switching mechanism to the OFF position (open loop position), which will forcibly disconnect the moving contact from the current-carrying parts.

The bimetal design shall be based on temperature/deflection requirements. Therefore, and for all cases, the bimetal should be within the given values in the time–current operating characteristics. For this reason, the discontinuity of the breakers (tripping) must be ensured that it has convenient circuit protection. Hence, the time–current operational characteristics define the tripping zone test of the bimetal where the rated current is applied to the circuit breakers with a specific time in several conditions that will locate whether the bimetal occurs within the limit or not and verify of bimetal desired deflection. However, all breaker conditions should be within the time–current zone specified by the standard IEC/EN 60898-1.

**Figure 2:**
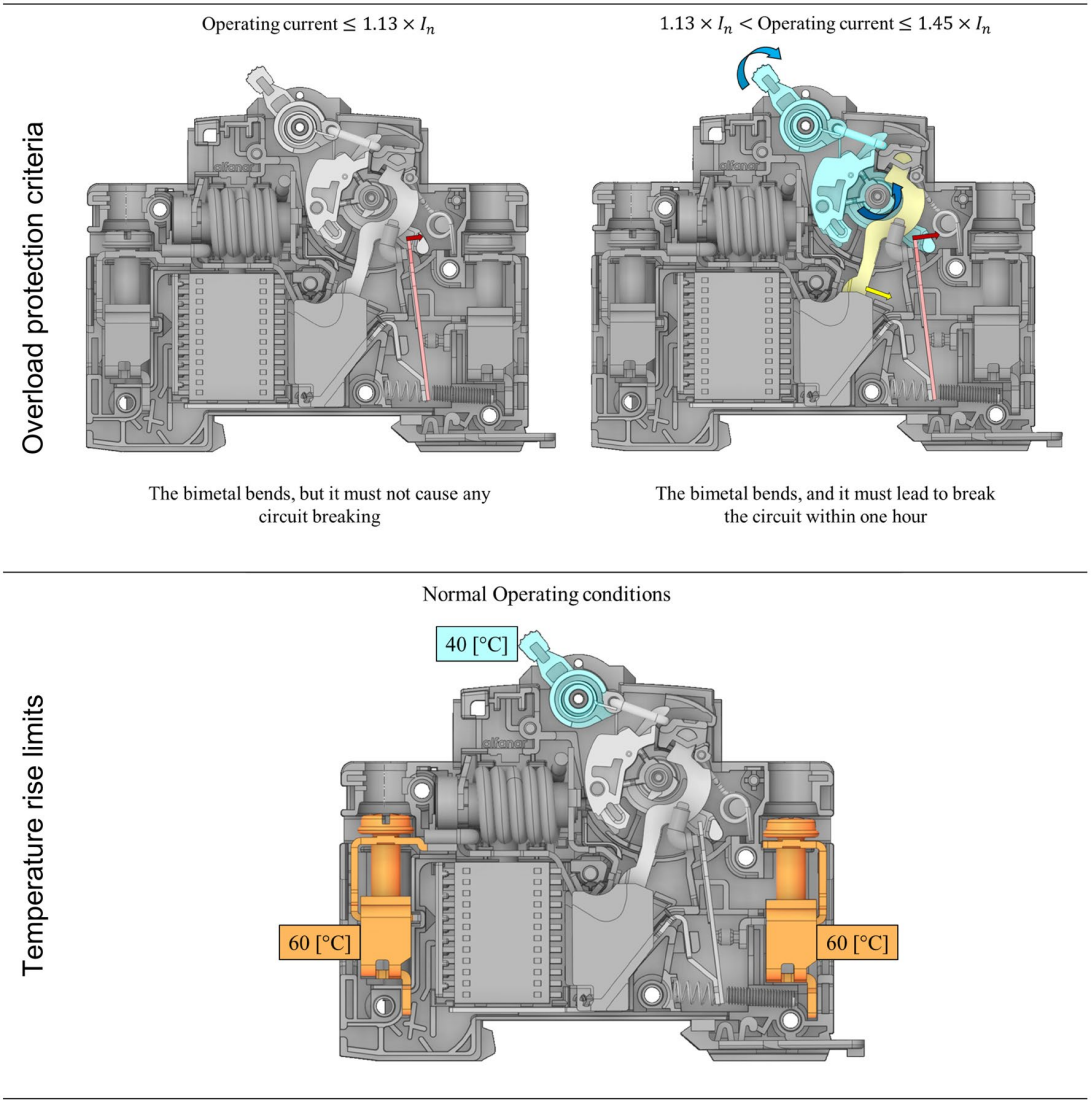
The requirements of IEC-60898-1 standard related to the bimetal design, genegrated using “ANSYS Spaceclaim R2 2021”^38^ (https://www.ansys.com/products/3d-design/ansys-spaceclaim).

The time–current test of the breakers must be carried out on calibrated breakers at a specified temperature which the manufacturer must declare. The purpose of the time–current test is to ensure the breaker in the closed position is safe and secured during any temperature rise (fault/overload condition). The temperature rise is the increase of the temperature inside the breaker, which will cause the deflection of the bimetal due to current overload that causes temperature exceeded the limit values specified by the standard. For instance, standard IEC/EN 60898-1 specifies the maximum temperature of 60 K for the terminals and other external parts, 40 K for the breaker handle (refer to Fig. 2 “generated using ANSYS Spaceclaim R2 2021”^38^) and 25 K for the external metallic parts of operating means.

Thus, a good bimetal design will affirmatively contribute to a relatively moderate temperature rise of the breaker. The material/composition selection will lead to determining the heat generated through the bimetal due to the resistivity of the material. As an overload protection device, a bimetal need to be utilized by applying force and displacement to trigger the tripping mechanism.

### Experiment

Special tests have been conducted to collect enough data to establish the ML model to consolidate the numerical model. The test of the effect of ambient temperature on tripping characteristics has a high and low temperature. The circuit breaker is placed ambient with a temperature of 25 ± 5 °C. The circuit breaker was connected to the power source using $2.5\, {\mathrm{mm}}^{2}, \, 4.0 \,{\mathrm{mm}}^{2}$ and $6.0\, {\mathrm{mm}}^{2}$ cables (based on the testing current), the current was passed through the product for 60 min. An RS PRO Type K Thermocouple 1/0.2 mm was connected to the center point of the bimetal, the thermocouple was also connected to a data logger (Yokogawa) to record the temperature during the test each 0.01 s. The test was carried out as per the instructions of the IEC-60898-1 standard (Fig. 2): the overload protection mechanism must break the circuit within one hour at the condition of $1.13\, \times\, {\mathrm{I}}_{\mathrm{n}}<$ Operating current $\le 1.45 \, \times \, {\mathrm{I}}_{\mathrm{n}}$, this criterion is directly related to the design of bimetal. The temperature rise of external parts must not exceed the limits defined in the IEC-60898-1 standard, as shown in Fig. 2.

### The machine learning model of the bimetal

After completing all the tests, the experimental results can be used as training samples for the ML model, training samples are a set of inputs and results, which have been experimentally obtained. For an n case of training samples, the input matrix gathers all the input parameters like ambient temperature (initial temperature), rated current, bimetal physical and thermal properties and bimetal dimensions. The final form of the input matrix for n training samples can be formulated^39^:

1A $${P}_{input}=\left[\begin{array}{l}{T}_{\mathrm{0,1}}^{P},{I}_{1}^{P},{V}_{1}^{P},{\gamma }_{1}^{P},{cp}_{1}^{P},{k}_{1}^{P},{\rho }_{1}^{P},{\varepsilon }_{1}^{P},{L}_{1}^{P},{W}_{1}^{P},{D}_{1}^{P},{T}_{{0,2}}^{P},{I}_{2}^{P},{V}_{2}^{P},{\gamma }_{2}^{P},\dots .,{T}_{0,i}^{P},{I}_{i}^{P},{V}_{i}^{P},{\gamma }_{i}^{P},\dots \\ \dots \dots ,{T}_{0,n}^{P},{I}_{n}^{P},{V}_{n}^{P},{\gamma }_{n}^{P},{cp}_{n}^{P},{k}_{n}^{P},{\rho }_{n}^{P},{\varepsilon }_{n}^{P},{L}_{n}^{P},{W}_{n}^{P},{D}_{n}^{P}\end{array}\right]$$

However, in this study, the problem's theoretical model can be derived using the energy balance method described in “Energy balance model”. The input matrix can be expressed as:

1B $${P}_{input}=\left[\begin{array}{l}{Q}_{g,1},{Q}_{cond,1},{Q}_{conv,1},{Q}_{rad,1},{\Delta U}_{1},{Q}_{g,2},{Q}_{cond,2},\dots .,{Q}_{g,i},{Q}_{cond,i},\dots \\ \dots \dots ,{Q}_{g,n},{Q}_{cond,n},{Q}_{conv,n},{Q}_{rad,n},{\Delta U}_{n}\end{array}\right]$$

The results matrix:

2 $${P}_{output}=\left[{T}_{bim,1}^{P},{T}_{bim,2}^{P},\dots .,{T}_{bim,i}^{P},\dots \dots ,{T}_{bim,n}^{P}\right]$$

**Figure 3:**
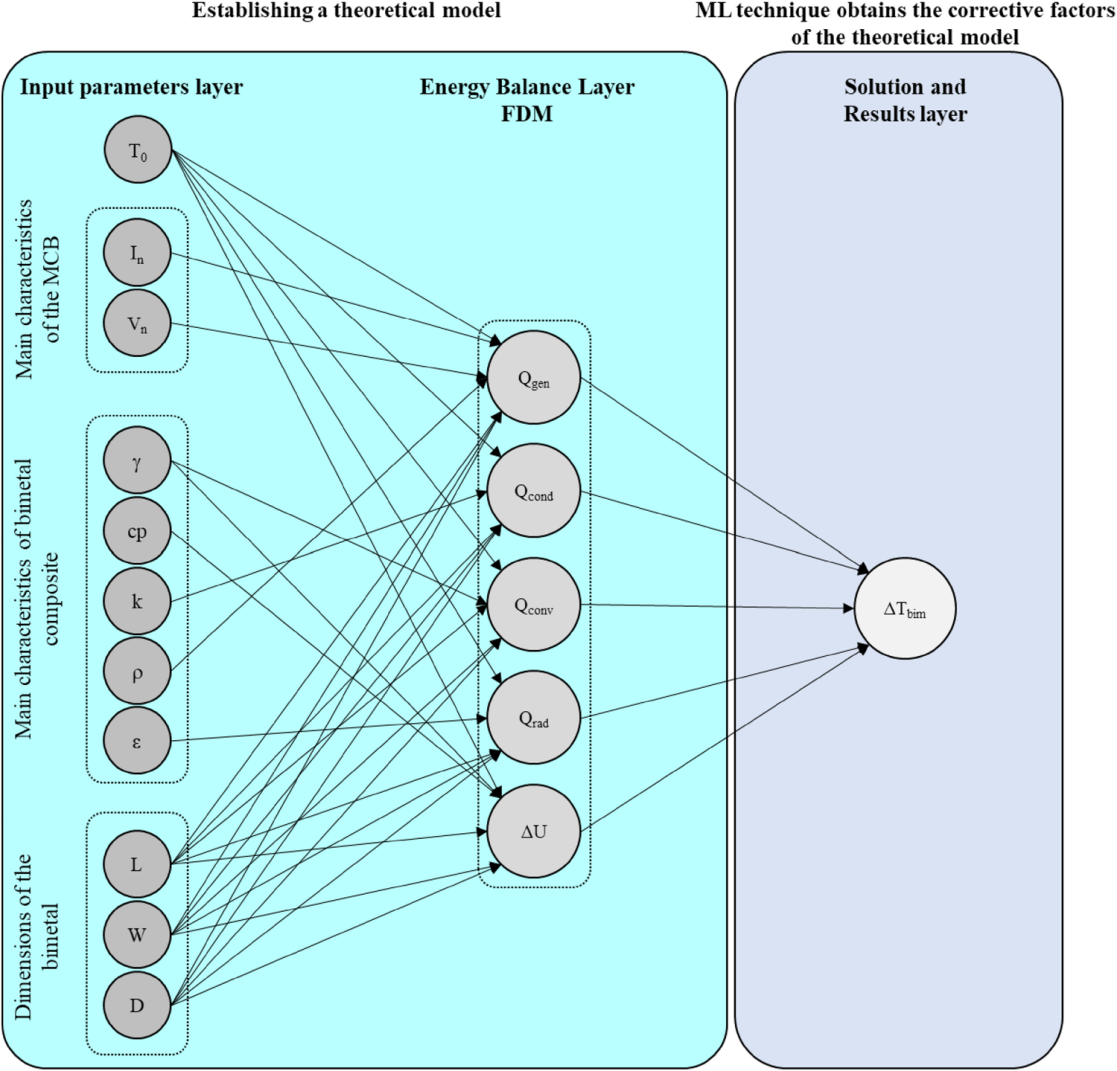
Neural network diagram of the machine learning model of bimetal inside the circuit breaker.

Generally, the role of the ML models is to find the relation between the input and output matrices^18,40^. But, in this work, the theoretical correlation is established and used to calculate the temperature rise of the bimetal since the relation between the input parameters and the temperature rise can be inferred (refer to “Energy balance model”). The energy balance model can be established based on the heat generation rate, thermal conduction, convection and radiation, after that, the model can be solved by the time-based finite difference method by considering all the input parameters. However, the theoretical model cannot address the effects of many other secondary parameters like the impact of assembling tens of components inside a small space, which crucially affects the convection heat transfer, the irradiation from other live parts, the heat transfer over the plastic parts in addition to many other factors. Hence, the ML method will be used to improve the theoretical model and correct the contribution of each thermal process to finally provide an accurate prediction about the temperature rise of the bimetal (Fig. 3). So, the NMML will be derived to finally get an accurate, fast, and easy model to be implemented for industrial applications.

### Energy balance model

The heat generation inside the conductor is defined as the squared current passing through the conductor ${I}^{2}$ multiplied by the resistance $R$:

3 $${Q}_{gen}={I}^{2}.R$$

When there is heat generation inside the conductor, the energy balance of the system can be expressed as:

4 $${Q}_{gen}-\left({Q}_{cond}+{Q}_{conv}+{Q}_{rad}\right)=\Delta U$$

where ${Q}_{gen}$ is the generated heat by the Joule heating effect, ${Q}_{cond}$ is the heat flux from the body to the connected bodies by conduction heat transfer, ${Q}_{conv}$ is the heat removed from the body to the surrounding fluid by convection, ${Q}_{rad}$ is the heat removed from the body to the surrounding environment by radiation, and $\Delta U$ is the change in the internal energy of the body. When the conductor resistance causes heat generation, the temperature of the body starts to increase from the temperature ${T}_{t}$ at the moment $(t)$ into the temperature ${T}_{t+\Delta t}$ at the moment $\left(t+\Delta t\right).$ The conduction heat transfer at the moment $(t)$ is given as:

5 $${Q}_{cond}={\eta }_{1}.\left(A.k.\frac{{T}_{t}-{T}_{con-B}}{\delta }\right)+{\xi }_{1}$$

Here, $A$ is the contact area where the bimetal is in touch with the conducting parts. Since the temperature of the connected body (${T}_{con-B}$) is unknown, and similarly, the thickness ($\delta$), another two regression coefficients ($\tau$ and $\theta$) can be added to the equation:

6 $${Q}_{cond}={\eta }_{1}.\left[A.k.\left(\tau .{T}_{t}+\theta \right)\right]+{\xi }_{1}$$

The convection heat transfer at the moment $(t)$ can be formulated as:

7 $${Q}_{conv}={\eta }_{2}.\left[{A}_{bim}.h.\left({T}_{t}-{T}_{amb}\right)\right]+{\xi }_{2}$$

where, ${A}_{bim}$ is the side area of the bimetal. The convection coefficient is expressed as:

8 $$h=\frac{k.Nu}{{L}_{bim}}$$

Nusselt number is strongly related to the orientation of the bimetal, i.e., whether it is mounted vertical or horizontal, can be obtained using the empirical correlations of the natural convection^41^:

9 $$Nu={\left\{0.825+\frac{0.387\times {Ra}_{bim}^{1/6}}{\left[1+\left(\frac{0.492}{Pr}\right)^{9/16}\right]{^{8/27}}} \right\}}^{2} \quad For\, the\, vertical\, plate$$

10 $$Nu=0.54\times {Ra}_{bim}^{1/4}\quad For\, the\, horizontal\, plate$$

For the inclined mounting, the incline angle must be considered in calculating the Nusselt number. Rayleigh and Prandtl numbers can be calculated by the correlations:

11 $$Pr=\frac{\vartheta }{\alpha }$$

12 $${Ra}_{bim}=\frac{g.\beta \left({T}_{t}-{T}_{amb}\right)}{{\vartheta }^{2}}.Pr$$

It is important to notify that $h$ is a function of the body temperature, ${T}_{t}$, since $Ra$ number is so. Hence, the convection coefficient must be recalculated for each time step. The radiation heat transfer at the moment $(t)$ can be expressed by the formula:

13 $${Q}_{rad}={\eta }_{3}.\left[A.\sigma .\varepsilon .\left({T}_{t}^{4}-{T}_{amb}^{4}\right)\right]+{\xi }_{3}$$

The internal energy change of the bimetal during a specified period, $\Delta t$, is correlated to the difference in the temperature during this period:

14 $$\Delta U=m.Cp.\left({T}_{t+\Delta t}-{T}_{t}\right)$$

Within a small period, any tiny change in the temperature of the bimetal $dT/dt$ causes a change in its internal energy $dU/dt$:

15 $$\frac{d{U}_{bim}}{dt}=m.Cp.\frac{d{T}_{bim}}{dt}$$

The energy balance equation of the bimetal can be formulated from Eqs. (3), (4), (6), (7), (13), (14) and (15) as:

16 $$\frac{dT}{dt}=\frac{{I}^{2}.R}{m.Cp}-\left\{{\eta }_{1}.\left[A.k.\left(\tau .{T}_{t}+\theta \right)\right]+{\eta }_{2}.\left[{A}_{bim}.h.\left({T}_{t}-{T}_{amb}\right)\right]+{\eta }_{3}.\left[A.\sigma .\varepsilon .\left({T}_{t}^{4}-{T}_{amb}^{4}\right)\right]+\xi \right\}/\left(m.Cp\right)$$

### Numerical model augmented by machine learning technique (NMML)

The time-based finite difference method can be used to simulate the temperature rise of the bimetal when an electric current passes through the circuit. Assuming that thermal homogeneously conditions are maintained at the moment t = 0, which implies the ambient conditions, the bimetal and all the components of the MCB have the same temperature. Once the current starts passing through the bimetal and due to its electrical resistance, a portion of the electric power is converted into heat as described by Eq. (3). Heat generation works to raise the temperature of the bimetal. Hence, the system becomes inhomogeneous and so, thermal losses represented by the thermal conduction, convection and radiation must work to reduce the temperature of the bimetal. To allow applying the Time-based forward finite difference approximation, time discretization into small time steps $\Delta t$ must be carried out. Equation (16) can be reformed to obtain the net temperature rise of the bimetal, the temperature at the new time step is then calculated by:

17 $${T}_{t+\Delta t}\approx {T}_{t}+\frac{\left\{\left({I}^{2}.R.\Delta t\right)-{\eta }_{1}.\left[A.k.\left(\tau .{T}_{t}+\theta \right)\right]-{\eta }_{2}.\left[{A}_{bim}.h.\left({T}_{t+\Delta t}-{T}_{fluid}\right)\right]-{\eta }_{3}.\left[A.\sigma .\varepsilon .\left({T}_{t}^{4}-{T}_{amb}^{4}\right)\right]+\xi \right\}}{m.Cp}$$

where $\xi ={\xi }_{1}+{\xi }_{2}+{\xi }_{3}$. In the case of bimetal inside MCB, the heat transfer process is very complicated because of the packing of many components within a small volume available in the MCB housing, some of these components are live parts that generate heat during the current passing, and others are electric and thermal isolators. Consequently, the air circulation by the natural convection heat transfer is distinctly restricted. Therefore, the theoretical formulas of conduction, convection, and radiation cannot accurately describe the temperature rise of the bimetal. To account for the actual heat transfer processes and their contribution to the energy balance equation, the regression coefficients $({\eta }_{1},{\eta }_{2},{\eta }_{3}, \xi ,\tau ,\theta )$ have been used with the finite difference model to correct the contribution weight of each heat transfer process.

After building the NMML model, and before it can be implemented, the regression coefficients $({\eta }_{1},{\eta }_{2},{\eta }_{3}, \xi ,\tau ,\theta )$ must be determined. For this purpose, the training matrices have been used. The solution of the NMML algorithm starts for each set of input parameters $(i)$ with the initial guess of the regression coefficients, and according to the initial values, the finite difference model (represented by Eq. 17) was solved, and the temperature of the bimetal for the new time step ${T}_{t+\Delta t}(i)$ has been determined. The solver continued calculating the temperature for each new time step until reaching the steady-state conditions when the temperature of the bimetal becomes stable:

18 $$If\left({T}_{t+\Delta t}(i)-{T}_{t}(i)=0\right) \, \to \,Steady \, state$$

Equation (18) represents the moment (t) where the bimetal reaches a steady-state condition. After obtaining the bimetal temperature for the case $(i)$, the solution can be continued for all of the $(n)$ cases in the training matrices. Then the cost function for the initial guess of the regression coefficients can be calculated employing the experimentally obtained temperature (${T}_{bim,i}^{P}$) and the theoretically calculated one (${T}_{t}\left(i\right)$)^39^:

19 $$J=\frac{1}{n}\sum_{i=1}^{n}{\left[{T}_{t}\left(i\right)-{T}_{bim,i}^{P}\right]}^{2}$$

**Figure 4:**
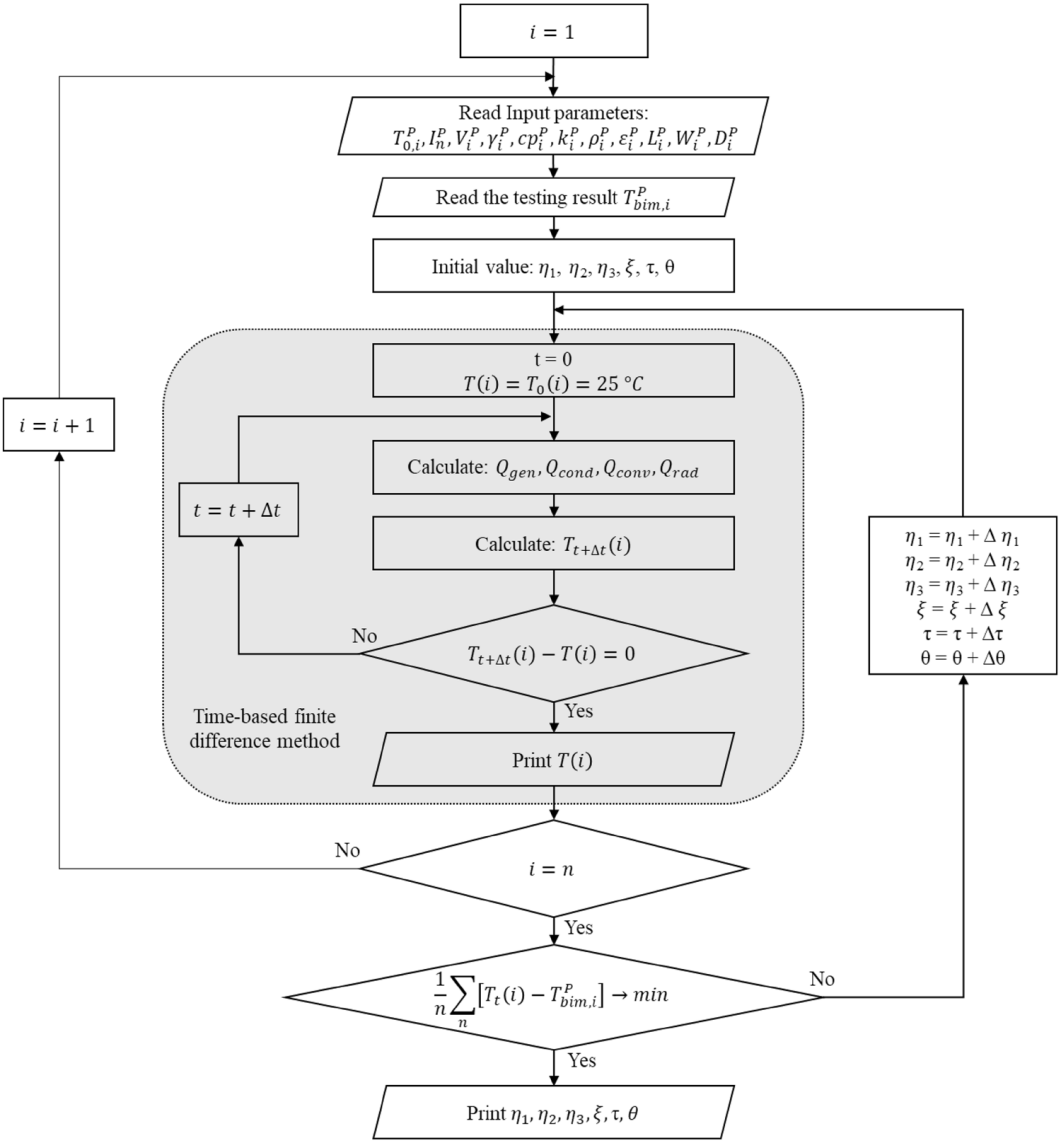
Algorithm flowchart of the NMML model.

The error function must be minimized by changing the values of the regression coefficients $({\eta }_{1},{\eta }_{2},{\eta }_{3}, \xi ,\tau ,\theta )$, the least square method was implemented^42,43^ in calculating the regression coefficients leading to minimizing the error function. The set of coefficients that fulfills the condition $\left(J\to min\right)$ are frozen for further utilization in solving practical problems. The above description is a brief presentation of the time-based finite difference method augmented by the ML model. The full method's schematic algorithm flow chart is illustrated in Fig. 4, where the algorithm was written in Python for practical utilization.

A set of $n=168$ training samples was provided for the model to obtain the regression coefficients $({\eta }_{1},{\eta }_{2},{\eta }_{3}, \xi ,\tau ,\theta )$, the training samples were obtained by a series of experiments as described in “Experiment”. The time step for the NMML has been chosen as 0.1 s and the analysis has been carried out for 3600 s (1 h) since the IEC 60898-1 standard specifies the tripping time due to the overload current with no more than one hour. The emissivity has been fixed for all types of the bimetal composites at 0.3 considering metal surface with a smooth surface^41^, whereas the suppliers of the bimetal provide other physical, thermal, and electrical properties. The ambient temperature has been taken from each experimental condition, and the dimensions of the bimetal are as per the design of the branch circuit breakers (MCB 16-63 A).

Seven types of bimetals listed in Table 2 with their physical properties were used in the experimental study. They were assembled in 28 sets of MCB products, each set has three breakers that have the same bimetal and were subjected to the same sets of testing currents (6 testing currents). Hence, the temperature rise of each bimetal for each testing current is the average of 3 readings, whereas the testing electric currents ranged from 8 to 91.4 A. The total number of training samples equals the number of testing sets 28 multiplied by the number of testing currents for each set 6. Hence, finally, 168 testing samples were being resulted from the experimental study, which will be used as training samples for the NMML model.

**Table 2:** Physical properties of the bimetals used in the experimental study.

Bimetal	BM-1	BM-2	BM-3	BM-4	BM-5	BM-6	BM-7
ρ (kg/m^3^)	8100	8200	8300	8300	8300	8400	8600
Cp (J/kg k)	460	460	460	440	440	420	400
k (W/m K)	13	18	22	28	70	125	250
ρ_e_ (Ω m)	$7.80\times {10}^{-7}$	$4.50\times {10}^{-7}$	$3.50\times {10}^{-7}$	$2.50\times {10}^{-7}$	$1.10\times {10}^{-7}$	$6.0\times {10}^{-8}$	$3.0\times {10}^{-8}$
$\varepsilon$	0.3	0.3	0.3	0.3	0.3	0.3	0.3
$\kappa$ (K^−1^)	$1.55\times {10}^{-5}$	$1.49\times {10}^{-5}$	$1.48\times {10}^{-5}$	$1.40\times {10}^{-5}$	$1.50\times {10}^{-5}$	$1.39\times {10}^{-5}$	$1.27\times {10}^{-5}$
E (MPa)	175,000	175,000	175,000	175,000	165,000	160,000	145,000
E_perm_ (MPa)	200	200	200	200	200	200	150
T_perm_ (C)	450	450	450	450	400	400	400

## Results

The experimental study was carried out for 168 cases, then, the results were used as training samples for the NMML model, as summarized in Fig. 4. Later, the regression coefficients have been obtained for the given MCB design. Finally, and after fixing the regression coefficients, the finite difference model has been used alone to evaluate the model. After the model, it can be utilized further in developing the bimetal to get the desired thermal tripping and reduce the temperature rise of the breakers under normal operations.

Based on the empirical study, training samples were prepared and fed into the NMML model. The novel NMML model was then ran to obtain the correlation between the thermal conduction, convection, and radiation with the heat generation due to the electric power passing the bimetal. The regression coefficients are shown in Table 3.

**Table 3:** Regression coefficients as obtained by the NMML model.

${\eta }_{1}$	${\eta }_{2}$	${\eta }_{3}$	$\xi$	$\tau$	$\theta$	${R}^{2}$
0.5	1.25	2	0	0.001	0.00225	0.99

The high value of ${R}^{2}$ implies a strong correlation between heat generation and heat transfer polynomials. By obtaining the regression coefficients, the model becomes ready for solving practical problems. The model must be validated initially by comparing it with testing results. A set of experiments was carried out using the rated currents and the bimetal physical and thermal properties in Table 4. The testing conditions were maintained as stipulated in the IEC 60898-1. The breakers were tested inside an oven, maintaining the ambient temperature at 50 ± 2 °C. The bimetal temperature was measured for each case and compared with the NMML model predictions.

**Table 4:** The details of Input parameters used for experimental validation of the NMML model.

Current (A)	ρ (kg/m^3^)	Cp (J/kg k)	k (W/m K)	ρ_e_ (Ω m)	ε	$\kappa$ (K^−1^)	E (MPa)
16	8100	460	13	$7.80\times {10}^{-7}$	0.3	$1.55\times {10}^{-5}$	175,000
18.1	8100	460	13	$7.80\times {10}^{-7}$	0.3	$1.55\times {10}^{-5}$	175,000
23.2	8100	460	13	$7.80\times {10}^{-7}$	0.3	$1.55\times {10}^{-5}$	175,000
20	8200	460	18	$4.50\times {10}^{-7}$	0.3	$1.49\times {10}^{-5}$	175,000
22.6	8200	460	18	$4.50\times {10}^{-7}$	0.3	$1.49\times {10}^{-5}$	175,000
29	8200	460	18	$4.50\times {10}^{-7}$	0.3	$1.49\times {10}^{-5}$	175,000
25	8300	460	22	$3.50\times {10}^{-7}$	0.3	$1.48\times {10}^{-5}$	175,000
28.3	8300	460	22	$3.50\times {10}^{-7}$	0.3	$1.48\times {10}^{-5}$	175,000
36.3	8300	460	22	$3.50\times {10}^{-7}$	0.3	$1.48\times {10}^{-5}$	175,000
32	8300	440	28	$2.50\times {10}^{-7}$	0.3	$1.40\times {10}^{-5}$	175,000
36.2	8300	440	28	$2.50\times {10}^{-7}$	0.3	$1.40\times {10}^{-5}$	175,000
46.4	8300	440	28	$2.50\times {10}^{-7}$	0.3	$1.40\times {10}^{-5}$	175,000
40	8300	440	70	$1.10\times {10}^{-7}$	0.3	$1.50\times {10}^{-5}$	165,000
45.2	8300	440	70	$1.10\times {10}^{-7}$	0.3	$1.50\times {10}^{-5}$	165,000
58	8300	440	70	$1.10\times {10}^{-7}$	0.3	$1.50\times {10}^{-5}$	165,000
50	8400	420	125	$6.0\times {10}^{-8}$	0.3	$1.39\times {10}^{-5}$	160,000
56.5	8400	420	125	$6.0\times {10}^{-8}$	0.3	$1.39\times {10}^{-5}$	160,000
72.5	8400	420	125	$6.0\times {10}^{-8}$	0.3	$1.39\times {10}^{-5}$	160,000
63	8600	400	250	$3.0\times {10}^{-8}$	0.3	$1.27\times {10}^{-5}$	145,000
71.2	8600	400	250	$3.0\times {10}^{-8}$	0.3	$1.27\times {10}^{-5}$	145,000
91.4	8600	400	250	$3.0\times {10}^{-8}$	0.3	$1.27\times {10}^{-5}$	145,000

**Figure 5:**
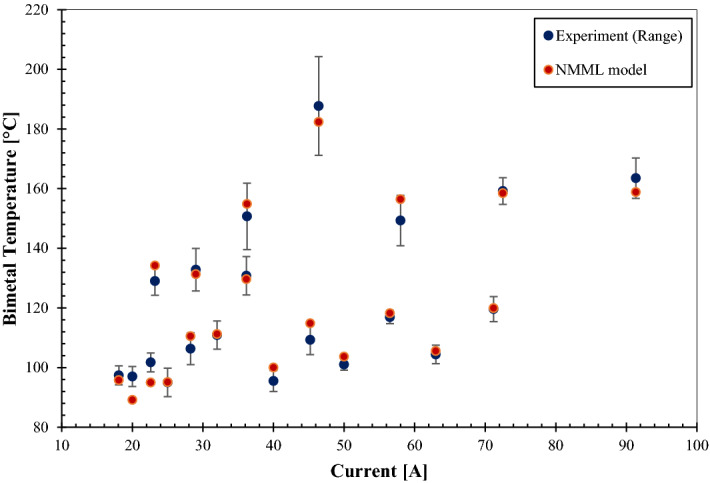
Validating the NMML model with experimental results.

Based on input parameters in Table 4, NMML-based numerical calculations have also been carried out. The results of experiments and numerical code are plotted in Fig. 5, the experimental readings are statistical since several readings were being taken for each case (minimum of three runs for each case). However, the NMML model predicts a single value for each case. Based on the comparison plotted in Fig. 5, the suggested NMML model has predicted 13 cases within the ranges given by the experiments, where 8 cases were out of the ranges. The maximum error of the novel code compared with the experimental results has not exceeded 8%, and the full range of experimental results for each given current has been considered for error calculation rather than the average value.

**Figure 6:**
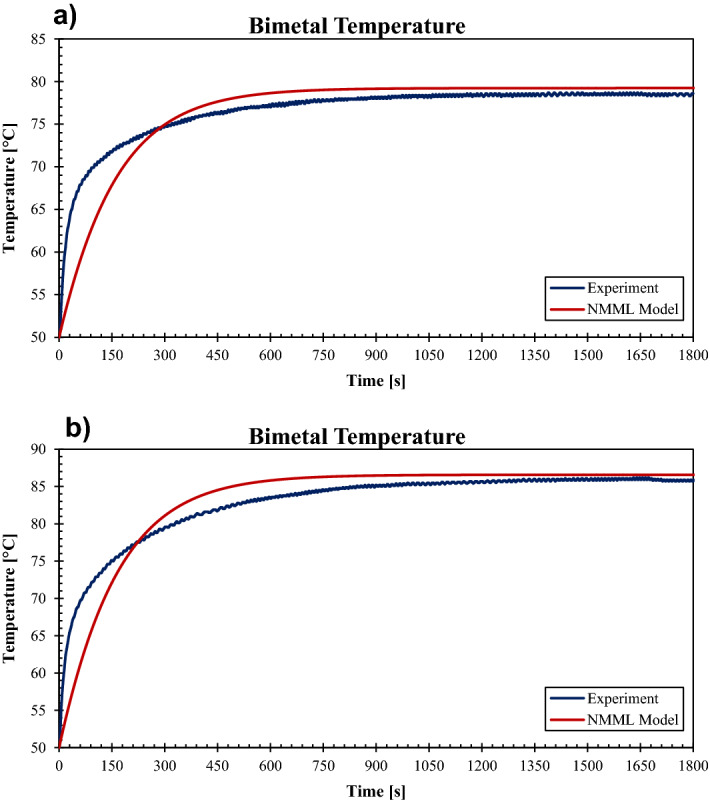
Bimetal temperature rise, experiment Vs. NMML model: (**a**) $I=18.8 \, \mathrm{A}$, BM-3*, *(**b**) $I=24.0 \, \mathrm{A}$, BM-4.

To shed more light on the thermal behavior of the bimetal, the temperature-rise profiles obtained experimentally and numerically have been compared to investigate the temperature development over time. Figure 6 reveals both practical measuring and numerical calculations of the temperature, give an exponential relation with the time. However, the measured bimetal temperature surges within a short period after passing the current, whereas the numerically obtained temperature gradually increases until the thermal balance with the surroundings approaches. The behavioral difference might be returned to the fact that the numerical model spontaneously calculates all the heat transfer processes for each time step, whereas the practical behavior takes a different route. For instance, the natural convection practically might take some time before being effective in the heat transfer process because of the restrictions on the air motion inside the breaker. Nevertheless, after the temperature remarkably increases inside the breaker, the breaker itself starts losing heat toward the environment. This is more likely the reason behind the fast surge up of temperature measured experimentally, in contrast to the simple natural convection model assumed for the NMML model.

**Figure 7:**
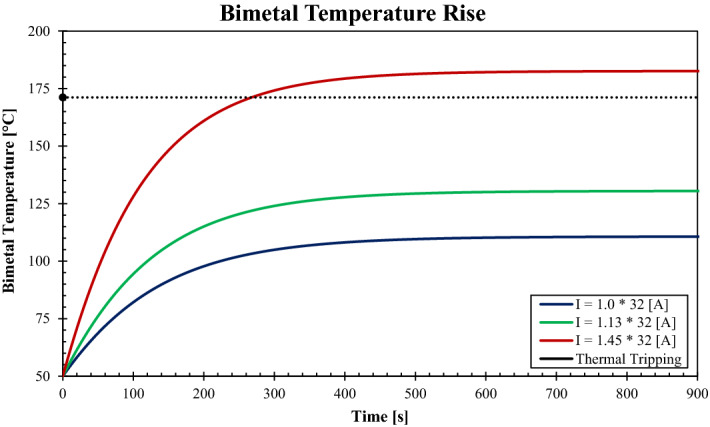
Temperature-rise of the BM-4 over the time for ${I}_{n}=32\, \mathrm{A}$ and other current conditions of $1.13\times {I}_{n}\,\mathrm{ and} \,1.45\times {I}_{n}$.

After fixing the regression coefficients and validating the NMML code, the model is ready to be utilized for predicting actual cases. The model was set to run for the rated current of 32 A, the standard requires investigating the behavior of bimetal for $1\times {I}_{n}, 1.13\times {I}_{n}\, \mathrm{and} \,1.45\times {I}_{n}$. The bimetal BM-4 with all properties as provided by the manufacturer (Auerhammer Metallwerk GmbH) has been used in the calculations. The bimetal BM-4 will be given the name BM-4, and its mechanical and thermal properties are shown in Table 5. In Fig. 7, the temperature rise profile of the bimetal in respect of time is plotted for the case of ${I}_{n}=32 \, \mathrm{A}$ and all required currents as declared by the standard. The temperature rise starts to increase dramatically when the current passes through the circuit breakers due to the heat generation. Nevertheless, after the bimetal temperature increase, the temperature rise rate gradually declines due to increasing heat losses. Finally, the bimetal temperature approaches the steady-state conditions, whereas the Joule heat generation equilibrates the thermal losses due to the conduction, convection, and radiation heat transfer processes into the surrounding environment. That explains why the temperature rise profile has an exponential relationship with time, the temperature rise profile is essential in designing the bimetal either for determining the dimensions of the part and/or choosing the proper composite. Obtaining the temperature of the bimetal is crucial not only for the system's safety but also for the temperature rise of the system. The excellent design considers the occurrence of thermal tripping when the overload current continues to pass through the circuit without causing a significant temperature rise either when running in normal conditions or the overload conditions. Indeed, the thermal tripping must happen within a reasonable time that does not exceed 3600 s (as stipulated by the IEC 60898-1 standard) the shorter the thermal tripping duration, the better and more sensitive design. The less the temperature rise required to achieve the thermal tripping conditions, the higher the efficiency indications of the invention.

**Table 5:** Mechanical and thermal properties of two bimetal composites: BM-4 and BM-8^29^.

Material	BM-4	BM-8
ρ (kg/m^3^)	8300	8200
Cp (J/kg k)	440	440
Thermal conductivity (W/m K)	28	50
Resistivity ( Ω m)	$2.5\times {10}^{-7}$	$1.5\times {10}^{-7}$
Radiation emissivity	0.3	0.3
Specific thermal curvature (K^−1)^	$1.4\times {10}^{-5}$	$2.8\times {10}^{-5}$
Max allowable temperature (°C)	450	400
Young modulus E (MPa)	175,000	165,000
Permissible stress E_perm_ (Mpa)	200	200

By obtaining the thermal behavior of the bimetal, the deflection can be determined when the bimetal temperature rises from ${T}_{1}$ to ${T}_{2}$ by applying the linear correlation^37^:

18 $${A}_{u}=\frac{0.53k\left({T}_{2}-{T}_{1}\right){L}^{2}}{s}$$

**Figure 8:**
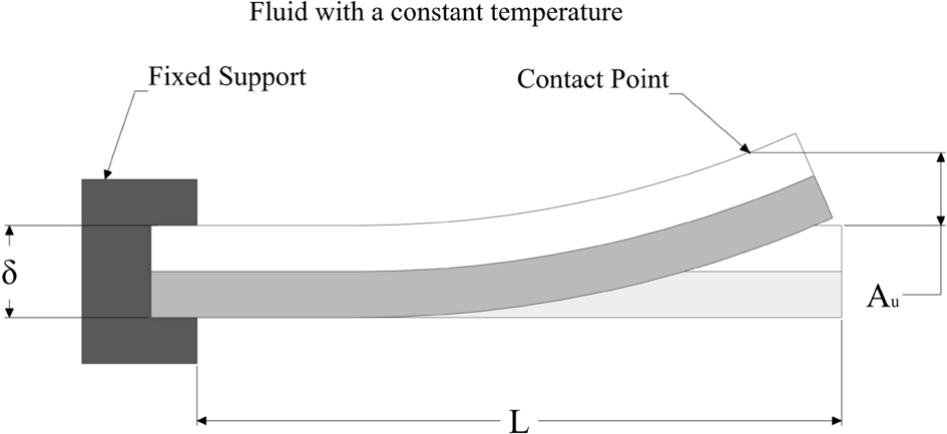
Deflection of the cantilever bimetal.

Increasing the bimetal temperature indeed leads to more deflection, the bimetal deflection is linked to the bimetal temperature by linear correlation. The bimetal continues deflecting since it does not touch the primary mechanism; this can be called a free deflection where the temperature-rise results only in a deflection. Figure 8 shows the case of free deflection of the cantilever bimetal fixed from one side where the second side is free to deform. All the geometry parameters contributing to Eq. (18) are clarified. Returning to Fig. 1, the cantilever bimetal used for the branch circuit breaker is fixed from the bottom and kept free to deflect from the top. Clearance has been also kept between the bimetal and the switching mechanism so, the bimetal can freely deform without causing any tripping for the normal operation. However, for a relatively high current estimated at $1.45\times {I}_{n}$, the bimetal deflection must be adequate to cover the entire clearance distance and apply the necessary force to switch off the mechanism. The calibration system is used to calibrate the clearance between the bimetal and the switching mechanism to allow the bimetal to cause tripping of the system when the current that passes directly through the bimetal is reached the threshold value.

**Figure 9:**
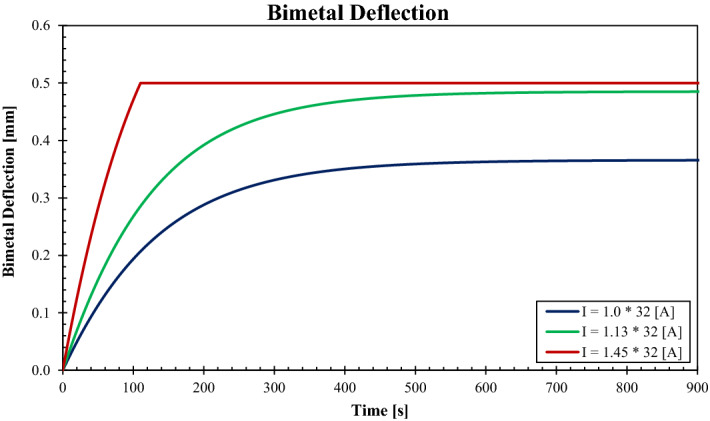
Deflection of the BM-4 over the time for ${I}_{n}=32\, \mathrm{A}$ and other current conditions of $1.13\times {I}_{n} \, \mathrm{ and} \, 1.45\times {I}_{n}$.

Once the bimetal temperature is obtained by the NMML model, the deflection is calculated using Eq. (18). The bimetal BM-4 with all properties as provided by the manufacturer (Auerhammer Metallwerk GmbH) has been used in the calculations. The case of rated current ${I}_{n}=32\, \mathrm{ A}$ and all the corresponding currents as stipulated in the IEC 60898-1 standard. The deflections for three currents are plotted in Fig. 9. Since the deflection has a linear relation with the bimetal temperature, the deflection profile is similar to the temperature. As the bimetal is free to deform, the exponential curve firmly describes how the bimetal responds to the temperature rise. Nevertheless, when bimetal touches the switching mechanism, the deflection profile reaches the maximum allowable value. After that limit, the effect of temperature rise alters by applying a force on the arm of the switching mechanism.

When the electric overload current passes through the circuit breaker, the bimetal responds by deflecting, until touching the switching mechanism. After that, the bimetal responds to the overload current by applying a bending force on the arm of the switching mechanism. The switching mechanism has mechanical resistance, where the switching mechanism breaks only if the required force is applied. Hence, the temperature of the bimetal must reach the tripping threshold to deflect and apply the tripping force. The bending force applied by the cantilever bimetal when it is fixed from one side and restrained from another side when its temperature rises from ${T}_{1}$ to ${T}_{2}$ can be calculated by the formula^37^:

19 $$F=\frac{0.53.k{s}^{2}bE({T}_{2}-{T}_{1})}{4L}$$

**Figure 10:**
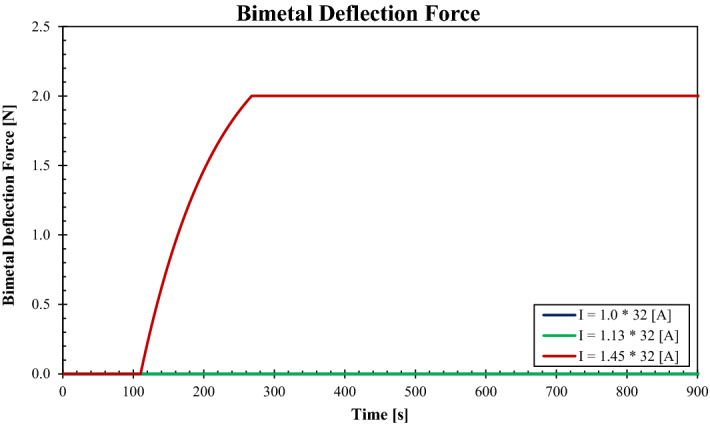
Deflection force applied by the BM-4 over the time for ${I}_{n}=32\, \mathrm{A}$ and other current conditions of $1.13\times {I}_{n}\, \mathrm{ and}\, 1.45\times {I}_{n}$.

The deflection forces have been calculated for the case of rated current ${I}_{n}=32 \, \mathrm{ A}$ and all the corresponding currents as stipulated in the IEC 60898-1 standard, and plotted in Fig. 10. The bimetal BM-4 with all properties, as provided by the manufacturer (Auerhammer Metallwerk GmbH) has been used in the calculations. As the results of the novel code depict that the specified bimetal is capable of applying the required force for tripping the mechanism and breaking the circuit when only the current passes through it exceeds the limit $I>1.13\times {I}_{n}$. For the case of ${I}_{n}$, the bimetal only deflects without closing the clearance with the primary mechanism. Nevertheless, for the case $1.13\times {I}_{n}$, the bimetal closes the gap with the switching mechanism but fails to reach the required tripping force. This is an excellent example of a design that complies with the requirements of the IEC 60898-1 standard.

## Discussion

In the previous sections, the utilization of the NMML model has been demonstrated in ensuring the design of bimetal and material selection comply with the IEC 60898-1 standard requirement and provides the desired functionality. Furthermore, the novel model can be extended to optimize the thermal performance of the circuit breaker product by helping in choosing the proper material for the given bimetal size to reduce the temperature rise of the bimetal without affecting its functionality, i.e., tripping at the electric current threshold of $I>1.13\times {I}_{n}$ within a relatively short time, that complies with the standard requirements. A series of numerical studies using the NMML model has been conducted to examine different combinations of the bimetal considering the manufacturer database to account the thermal and mechanical properties. Based on the results dramatic reduction in the temperature has been achieved by changing the existing material (bimetal BM-4) with a new BM-8 as given by Auerhammer Metallwerk GmbH^37^. The mechanical and thermal properties of both bimetals are given in Table 5.

**Figure 11:**
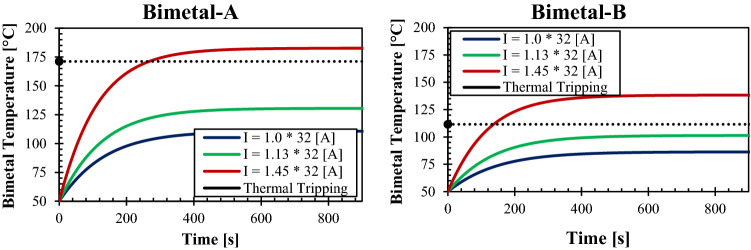
A comparison of BM-4 and BM-8 (Auerhammer Metallwerk GmbH) bimetals under the same conditions of electric currents, equal size and exact interfacing parameters with the switching mechanism of the branch circuit breaker 32 A.

Based on the examination study of different materials, the BM-8 was found to exhibit promising results that it provides the required functionality by tripping only when the condition $I>1.13\times {I}_{n}$ is fulfilled. Further, the BM-8 generates less heat, causing a lower temperature rise rate than the BM-4. A comparison of BM-4 and BM-8 (Auerhammer Metallwerk GmbH) bimetals under the same conditions of electric currents, equal size, and exact interfacing parameters with the switching mechanism of the branch circuit breaker 32 A is plotted in Fig. 11. Additionally, the BM-8 is more susceptible to the temperature rise that deflects with a significantly large rate even at a relatively moderate temperature rise. Hence, it works outstandingly for the branch circuit breaker with a rated current of 32 A. The temperature of the BM-8 at normal conditions ($I=32 \, \mathrm{A}$) reaches around $86\, ^{\circ}{\rm C}$ comparing to around $110 \, ^{\circ}{\rm C}$ for the BM-4. Therefore, there is a $24 \, ^{\circ}{\rm C}$ difference between the two materials when running under the same conditions. The bimetal, generally, generates the maximum amount of heat inside the circuit breaker since its resistivity is remarkably higher compared to all other current-carrying parts. Hence, it is most likely to possess the maximum temperature inside the circuit breaker. Choosing the right material indeed must consider the requirements of the IEC 60898-1 standard for temperature rise, which must not exceed 60 K for the terminals and other external parts, 40 K for the breaker handle and 25 K for the external metallic parts.

The BM-8 provides superb characteristics in terms of thermal tripping and operating under normal conditions, and both aspects guarantee the safe operation of the product. Apart from the normal operation conditions, the BM-8 thermally trips at the temperature of $109 \, ^{\circ}{\rm C}$, whereas the BM-4 thermally trips at the temperature of $168 \, ^{\circ}{\rm C} .$ This noticeable temperature difference provides another safety aspect for considering the BM-8 in the design since it trips before approaching severe temperatures. Table 6 provides a comprehensive comparison between the two materials; the results have been obtained using the novel NMML model. Based on the results, the BM-8 trips within only 181 s compared to 287 s for the BM-4. The fast response of the BM-8 adds an extra feature to use instead of the BM-4. Finally, both bimetals apply the required thermal tripping force; however, the results show a slightly higher force for the BM-8, this is due to the accuracy of the model. By using a more minor time step lower than 0.1 s, the tripping force of the two materials becomes almost the same.

**Table 6:** A comparison of the performance of BM-4 and BM-8 bimetals under the same conditions.

	BM-4	BM-8
The bimetal temperature rise under normal operation (°C)	110.9	86.4
The bimetal temperature at tripping conditions (°C)	168.1	109.9
Tripping current	1.13 × In < I_trip_ < 1.45 × In	1.13 × In < I_trip_ < 1.45 × In
Tripping time (s)	287	181
Deflection force at tripping condition (N)	4.95	5.05

Referring to other methods of studying different materials for this application, there are mainly two major methods that can be used to investigate the temperature rise of the bimetal in CB products. The first one is the experimental method, where many types of bimetals can be tested under different conditions to determine their functionality for the given size available inside the circuit breaker. However, there are many difficulties when running experiments for this purpose, one related to fulfilling the requirements of thermal tripping and a relatively low temperature during the normal operations required to examine a lot of bimetal composites to determine their validity for each rated current. Additionally, the required time to procure the material and samples and then run the tests, makes this method is not applicable for many cases that linked to a firm development plan and time frame like designing a new range of circuit breaker products. Another method to study the temperature rise problem for the bimetal is the numerical approach, where finite element methods and others can be implemented to examine the products. Nonetheless, there are also several restrictions and drawbacks of depending on the numerical methods. One related to the required time to prepare and run the model, which takes several weeks or even months to investigate a wide range of the bimetal composites for different rated currents and conditions. Further, numerical models cannot provide accurate predictions of the temperature rise since the circuit breaker product is packed with dozens of components of different materials by considering the effects of all components on the temperature rise of the bimetal, additional complications will be added to the model, and so, extra time will be required to solve each case. That indeed makes the thermal phenomena inside the product very sophisticated to be modeled by the conventional methods and so, difficult to get accurate results. The accuracy of the model is crucial for this application since the given problem is about determining certain values of the temperature rise under different conditions; failing the accurate obtain the thermal behavior of the bimetal may seriously affect the safety of the product.

Apart from the conventional methods, the novel NMML model provides a compensation methodology that exploits the advantages of both experimental and numerical approaches. The NMML model exhibits a good accuracy that satisfies the industrial needs and fulfills the product's safety requirements. Moreover, the model is fast and robust when running throughout the full range of the rated currents, it can immediately provide comprehensive information about the temperature rise profile of the bimetal and many other performance curves once the material properties and the model dimensions are provided. Thanks to the numerical finite difference method, which helps to obtain the deterministic performance parameters for each time step till the reaching the thermal balance. Hence, a vast spectrum of bimetal composites can be examined within a very short time to choose the best material capable of performing perfectly for each rated current. Furthermore, the required testing samples are not so large since only a few bimetal composites can be tested under different rated currents to determine all the model's regression coefficients. The aspects of reliability and applicability rightly fit with the industrial needs, the NMML model is simple to build and run, besides its robustness and reliability.

ML mode is usually used alone to find the correlations between different factors affecting the desired phenomenon^44^. Nevertheless, the theoretical correlation in this study has been established for the heat transfer in the MCB products, and then, ML model was implemented to reduce the error of the theoretical model. To manifest the role of ML in the suggested NMML model, the temperature rise of the bimetal is studied in two methods: the first one was performed by only the NM, whereas the hybrid NMML did the second method. The results of both methods along with the empirically measured temperature rise are shown in Table 7 for different conditions. The standard error of the empirical study is obtained, so the errors of ML and NMML are also determined. The comparison shown in Table 7 unveiled that NM alone cannot provide accurate predictions of the bimetal thermal behavior since the error is significantly large. The NM alone is unreliable for such a complicated heat transfer process in the MCB since the error is usually higher than 15.8%, in fact, the NM results in a very high error that exceeds 50% in many cases. In contrast, the NMML has a relatively low error that does not exceed 8.1%, thanks to the ML model for addressing the influence of several factors that could not be considered in the theoretical frame (like the convection inside a compact space, the contribution of different metal and plastic parts into the heat transfer, etc.). The empirical measurement has a limited accuracy with a standard error reaching 8.8%. The standard error of the experiment can be explained by the complex assembly of many parts with tolerances in distances, parts’ dimensions, and compositions, besides the measurement errors. On the other hand, the number of required learning samples is relatively low since the main factors influencing the heat transfer in the bimetal is formulated by the theoretical model. Therefore, constructing the NMML model is significantly easier than building only an ML model without the base of the energy balance model.

**Table 7:** The standard error of the experimental results, the calculation error of the NM and NMML models.

I (A)	Bimetal	Experimental		NM		NMML	
		T_Experimental_ (°C)	Standard error (%)	T_NM_ (°C)	NM error (%)	T_NMML_ (°C)	NMML error (%)
18.1	BM-1	97.4	3.3	82.0	15.8	95.8	1.6
20.0	BM-2	97.0	3.7	66.8	31.1	89.2	8.1
22.6	BM-2	101.7	3.5	71.5	29.7	95.0	6.7
23.2	BM-1	129.0	3.1	102.4	20.6	134.2	4.0
25.0	BM-3	95.0	5.4	66.9	29.5	95.1	0.1
28.3	BM-3	106.3	5.0	71.6	32.6	110.5	4.0
29.0	BM-2	132.8	5.0	85.2	35.8	131.3	1.2
32.0	BM-4	110.9	7.4	65.8	40.7	111.3	0.3
36.2	BM-4	130.8	4.2	70.1	46.4	129.6	0.9
36.3	BM-3	150.7	4.9	85.5	43.3	154.9	2.8
40.0	BM-5	95.5	8.8	54.4	43.0	100.0	4.7
45.2	BM-5	109.3	3.7	55.7	49.1	114.8	5.1
46.4	BM-4	187.7	4.6	83.1	55.7	182.3	2.9
50.0	BM-6	101.0	5.7	52.1	48.4	103.7	2.6
56.5	BM-6	116.9	1.8	52.7	54.9	118.2	1.1
58.0	BM-5	149.3	1.9	59.4	60.2	156.4	4.8
63.0	BM-7	104.4	2.8	50.8	51.3	105.5	1.1
71.2	BM-7	119.6	3.0	51.1	57.3	119.9	0.3
72.5	BM-6	159.2	3.5	54.5	65.8	158.5	0.5
91.4	BM-7	163.5	4.1	51.8	68.3	158.8	2.9

## Conclusion

In this study, a novel NMML model has been built to predict the thermal performance of the bimetal inside the electrical circuit breaker products. The conventional methods cannot provide an accurate and rapid solution for the problem of selecting the right material and size for the bimetal in the MCB products to fulfill the thermal tripping requirements and a relatively low-temperature rise under normal operating conditions. The suggested model is based on a numerical method augmented with a ML model. The numerical method of the time-based finite difference method marks the theoretical frame of the component, whereas ML model gives the advantages of the experimental results to correct and make the theoretical model more accurate. The novel NMML model has been built based on:

- Numerical method: time-based finite difference method to theoretically frame the energy balance of the bimetal during the electric current passing through the circuit breaker.
- ML model: to obtain the corrective factors of the theoretical frame and improve the accuracy of the model. An experimental study has been conducted to get the training samples. then, the NMML model was trained to approximate the heat transfer processes and finally get the temperature rise of the bimetal.
- The novel NMML model has been validated with the experiment study, it exhibits a good agreement with the empirical results. The maximum error does not exceed 8% when the model has been validated for different materials and rated currents.
- The model has been used to predict how the bimetal performs in the circuit breaker under different conditions. The temperature rise profile shows an exponential relation over time, the maximum temperature of the bimetal after reaching the thermal balance conditions can be obtained, along with the required time for the thermal equilibrium can be obtained by the model. With obtaining the temperature rise profile, the deflection of the bimetal and the deflection force applied by the bimetal on the switching mechanism can both be determined over time. Hence, comprehensive information under different conditions can be obtained by the model.
- The model has been used to optimize the material of the bimetal, for instance, the maximum temperature inside the branch circuit breaker 32 A has been reduced by $24 \,^{\circ}{\rm C}$ when the model is used to choose between different bimetal composites. Further, the suggested material responds fast to the temperature change by a higher deflection rate. Hence, the new material shows a remarkably lower time for thermal tripping. This depicts how this model can be utilized to improve the safety and operation conditions of the breaker.
- The NMML significantly improves the accuracy of the numerical model; the error of NM exceeds 50%, but the NMML model reduces the error to below 8.1. The standard error of the experiment reaches 8.8%. However, the numerical model in this study was performed over one element.
- The NMML is a promising model. In contrast to the conventional ML methods, the relation that links the influential factors of a specified phenomenon is theoretically determined. The role of ML in the suggested hybrid method is only to improve the model's accuracy. Hence, the number of training samples is significantly reduced. On the other hand, the numerical model is made of only one element that makes solving the model fast. Therefore, the NMML model is fast and accurate for engineering applications that require numerous iterations of experiments or numerical solutions (Supplementary information).

## Supplementary Information

Supplementary Information.

## Data Availability

The data used in the current research is available in the submission.

## List of symbols

$$A$$: Area (m^2^)
${A}_{u}$: The deflection of the bimetal plate due to the heating (mm)
$b$: The width of the bimetal plate (mm)
$Cp$: Specific heat (J/kg K)
$E$: Modulus of elasticity (MPa)
$$F$$: Force (N)
$g$: Acceleration of gravity, 9.8066 (m^2^/s)
$h$: Coefficient of convection heat transfer (W/m^2^ K)
$$J$$: Error function
$$k$$: Thermal conductivity (W/m K)
$L$: Active length (mm)
$Nu$: Nusselt number, non-dimensional number
$Pr$: Prandtl number, non-dimensional number
$Q$: Heat flow rate (W)
$$R$$: Electric resistance (Ω)
$Ra$: Rayleigh number, non-dimensional number
$$s$$: Thickness (mm)
$$T$$: Temperature (K)
$$t$$: Time (s)
$$U$$: Internal energy (J)
$$V$$: Volume (m^3^)

## Greek

$$\alpha$$: Thermal diffusivity (m^2^/s)
$\beta$: Thermal expansion coefficient (1/C)
$$\delta$$: Thickness (m)
$$\varepsilon$$: Emissivity
$\kappa$: Specific thermal curvature (1/C)
$$\rho$$: Density (kg/m^3^)
$${\rho }_{e}$$: Electric resistivity (Ω m)
$\sigma$: Stefan–Boltzmann constant, 5.67 × 10^–^^8^ (W/m^2^ K^4^)
$$\vartheta$$: Viscosity (m/s^2^)

## Subscripts

$${}_{0}$$: Initial condition
$${}_{amb}$$: Ambient
$${}_{bim}$$: Bimetal
$${}_{con-B}$$: Connected body
$${}_{cond}$$: Thermal conduction
$${}_{conv}$$: Thermal convection
$${}_{fluid}$$: Surrounding fluid
$${}_{gen}$$: Heat generation
$${}_{rad}$$: Thermal radiation

## Abbreviations

FDM: Finite difference method
MCB: Miniature circuit breaker
ML: Machine learning
NMML: Numerical model augmented by machine learning
